# Air filtration mitigates aerosol levels both during and after endoscopy procedures

**DOI:** 10.1002/deo2.231

**Published:** 2023-04-17

**Authors:** Frank Phillips, Jane Crowley, Samantha Warburton, Karren Staniforth, Adolfo Parra‐Blanco, George S.D. Gordon

**Affiliations:** ^1^ NIHR Nottingham Biomedical Research Centre Nottingham University Hospitals NHS Trust and the University of Nottingham Nottingham UK; ^2^ Department of Electrical and Electronic Engineering University of Nottingham Nottingham UK; ^3^ NIHR Nottingham Biomedical Research Centre Nottingham University Hospitals NHS Trust and the University of Nottingham Nottingham UK; ^4^ UK Health Security Agency Seaton House City Link Nottingham UK; ^5^ NIHR Nottingham Biomedical Research Centre Nottingham University Hospitals NHS Trust and the University of Nottingham Nottingham UK

**Keywords:** aerosols, air conditioning, COVID‐19, endoscopy, infection control

## Abstract

**Objectives:**

Upper gastrointestinal endoscopies are aerosol‐generating procedures, increasing the risk of spreading airborne pathogens. We aim to quantify the mitigation of airborne particles via improved ventilation, specifically laminar flow theatres and portable high‐efficiency particulate air (HEPA) filters, during and after upper gastrointestinal endoscopies.

**Methods:**

This observational study included patients undergoing routine upper gastrointestinal endoscopy in a standard endoscopy room with 15–17 air changes per hour, a standard endoscopy room with a portable HEPA filtration unit, and a laminar flow theatre with 300 air changes per hour. A particle counter (diameter range 0.3 μm‐25 μm) took measurements 10 cm from the mouth. Three analyses were performed: whole procedure particle counts, event‐based counts, and air clearance estimation using post‐procedure counts.

**Results:**

Compared to a standard endoscopy room, for whole procedures we observe a 28.5x reduction in particle counts in laminar flow (*p* < 0.001) but no significant effect of HEPA filtration (*p* = 0.50). For event analysis, we observe for lateral flow theatres reduction in particles >5 μm for oral extubation (12.2x, *p* < 0.01), reduction in particles <5 μm for coughing/gagging (6.9x, *p* < 0.05), and reduction for all sizes in anesthetic throat spray (8.4x, *p* < 0.01) but no significant effect of HEPA filtration. However, we find that in the fallow period between procedures HEPA filtration reduces particle clearance times by 40%.

**Conclusions:**

Laminar flow theatres are highly effective at dispersing aerosols immediately after production and should be considered for high‐risk cases where patients are actively infectious or the supply of personal protective equipment is limited. Portable HEPA filers can safely reduce the fallow time between procedures by 40%.

## INTRODUCTION

Following the coronavirus disease 2019 pandemic, it has now been proven that upper and lower endoscopic procedures can generate aerosols, although their infectivity by severe acute respiratory syndrome coronavirus 2 (SARS‐CoV‐2) remains unclear, especially for lower gastrointestinal (GI) endoscopy.^1^, ^2^ There have been various approaches to mitigating aerosols during endoscopy, including the use of facemasks on patients, which is increasingly being adopted clinically,^3^, ^4^ and the substitution of per‐oral for trans‐nasal endoscopy, which can reduce aerosol production by 50%.^1^ Improved ventilation is another key aerosol mitigation strategy but recommendations vary widely: newly designed endoscopy rooms in the UK require at least 10 air changes per hour (ACH), and negative pressure.^5^ Operating theatres with laminar air flow are widely used as means of limiting airborne transmission of pathogens and contaminants.^6^ For example, they have been shown to reduce aerosol concentration by factors of 100 or more in arthroplasty.^7^ Laminar flow theatres are therefore a promising approach to mitigate aerosols during digestive endoscopy. Gregson et al. measured particle counts during upper digestive endoscopy in an operating theatre with the laminar flow but during measurements the ventilation was set to ‘standby’, reducing the ACH from 500–600 to 25.^8^ Previous work has shown that reducing ACH to 25 does not significantly reduce particles measured from volitional coughing, but reducing ACH to zero dramatically increases particles.^9^ However, no comparison has been performed with ventilation conditions in typical endoscopy rooms without laminar flow and whose ACH is typically lower than a laminar flow theatre on standby, nor have typical events encountered in endoscopy (involuntary gagging, extubation, throat spray) been characterized under these conditions. The mitigating effect of fully operative laminar flow on aerosol levels during digestive endoscopy compared to typical endoscopy rooms has therefore not been assessed.

Portable high‐efficiency particulate air (HEPA) filtration units are a lower‐cost alternative to laminar flow systems for mitigating aerosols. Numerous studies have shown the ability of a range of portable HEPA units to remove aerosols from the air,^10^ but these have mostly been conducted under controlled laboratory conditions. More recently, clinical studies in intensive care units showed that portable HEPA filtration units can significantly reduce viable SARS‐CoV‐2 in air samples.^11^, ^12^ The use of portable HEPA filtration has been proposed for use in endoscopy units^13^ but there have been no clinical studies on the effect under typical procedure conditions.

## METHODS

The methodology used for this study is based on what we developed for a previous “baseline” study of aerosol generation in digestive endoscopy.^1^ Health Research Authority and ethical approval were granted by the Wales Research Ethics Committee prior to the start of the study (IRAS no. 285595). We included patients undergoing routine upper GI endoscopy on the lists of thirteen different participating endoscopists at the Endoscopy Unit of the Nottingham University Hospitals NHS Trust Treatment Centre between October 2020–March 2021. The inclusion criteria were adult patients >18 years with the capacity to consent. Procedures were performed as they normally would be in clinical practice.

### Experimental procedure

**FIGURE 1:**
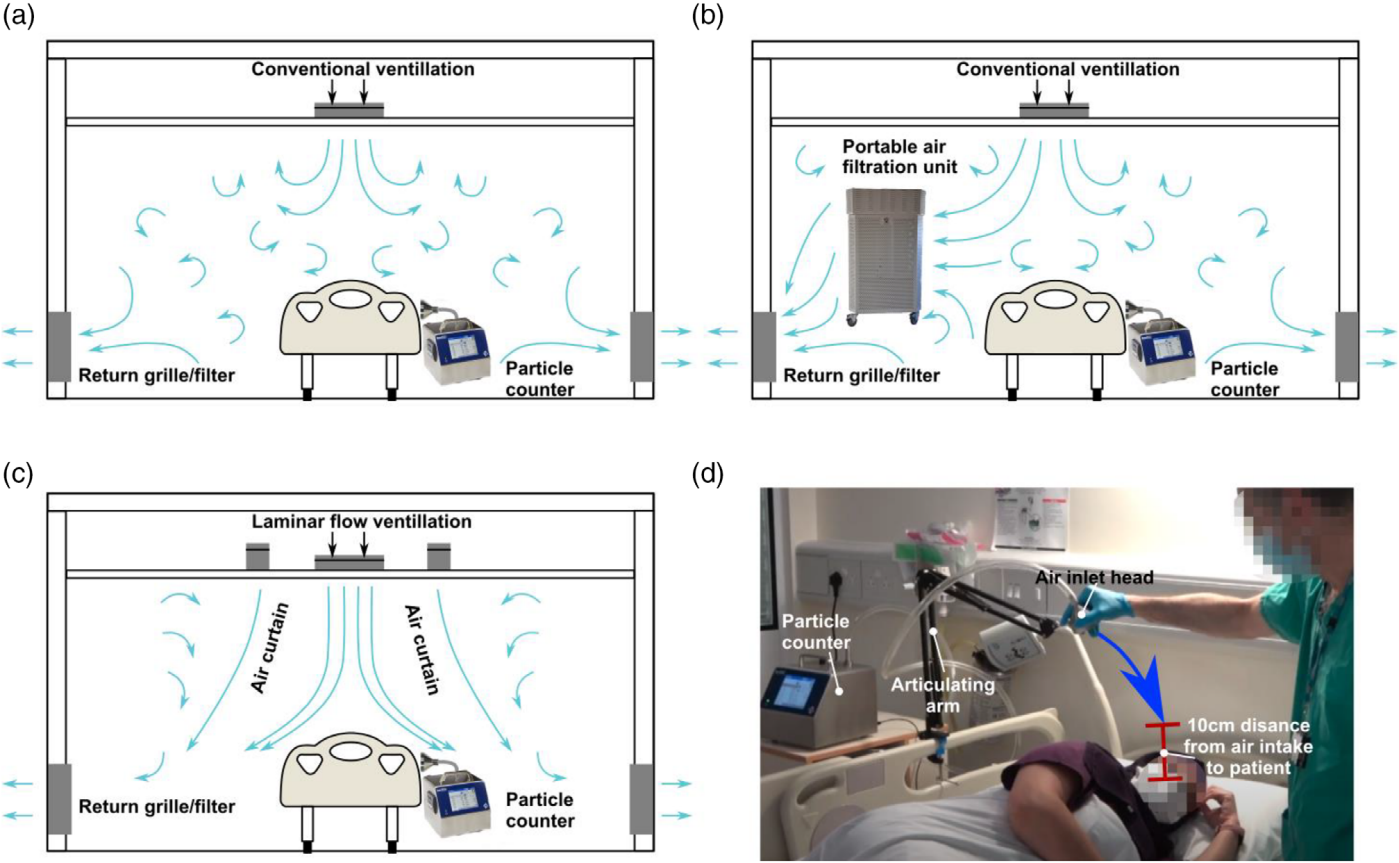
Schematic showing different ventilation conditions and measurement set‐up: (a) conventional ventilation with typically weaker flow and re‐circulating air currents, (b) conventional ventilation with portable high‐efficiency particulate air filtration unit acting to increase the rate of air filtration and slightly increase air flow, (c) laminar flow ventilation which creates powerful curtains of air to quickly remove all particles creating an ultra‐clean environment, and (d) photograph showing typical measurement configuration with air‐inlet to particle counter place on articulating arm and lowered to within 10cm of the patient's mouth.

We measured the concentration of aerosols (<5 μm diameter) and droplets (>5 μm diameter) produced during typical upper GI endoscopy procedures conducted both in standard endoscopy rooms (*n* = 33), standard endoscopy rooms with portable HEPA filtration units (Air Sentry Limited, Wiltshire, UK; *n* = 4) and in laminar flow theatres (*n* = 4 full procedures, *n* = 9 for volitional cough and throat spray). These three different ventilation scenarios and typical airflow patterns are shown in Figure 1a–c, respectively. Ventilation effectiveness is affected by room size and placement of air filtration units but, for reasons of practicality, we consider a limited subset that represents a typical clinical scenario. Specifically, all rooms used are in the same endoscopy unit with similar ventilation (15–17 ACH, measured using a balometer), size, air temperature, and humidity levels. Only a single room was available for the HEPA filtration unit but it was professionally installed by the supplier and so presumably exhibits near‐peak performance. The laminar flow theatres have much higher ventilation (300 ACH) and are, by necessity, of different design to conventional endoscopy rooms but are representative of a practical mitigation strategy that we wish to examine. All procedures considered are upper GI endoscopy procedures, which we previously showed are AGP.^1^

Particle counts were measured and analyzed using an AeroTrak portable particle counter (model 9500–01; TSI, Shoreview, MN, USA) with an isokinetic inlet head placed 10 cm from the patient's mouth via a 2 m tube (manufacturer provided), shown in Figure 1d. Previous studies have shown that detecting particles at distances greater than 10 cm from the source dramatically reduces detectable particle count and biases particle size.^9^ For this reason, and for comparability with previous studies,^2^ a distance of 10cm is chosen. The patient's mouth is considered the main source of potentially infectious particles since all other persons in the room are wearing FFP3‐grade (N95) masks for this study. The particle counter measures particle counts in six diameter ranges (0.5–0.7, 0.7–1.0, 1.0–3.0, 3.0–5.0, 5.0–10.0, and 10.0–25 μm) and has a flow rate of 100 L/min, with readings taken every 7s. The effect of the tube length on larger particles is accounted for by calibration prior to the experiment.

We compared aerosol and droplet concentrations produced from whole procedures (median duration of 7.2 min), but we normalize counts to a 20‐min procedure by multiplying the total particle count by an appropriate factor. Sedation conditions vary across patients from only using Xylocaine throat spray to the use of Midazolam. However, previous studies have not found a significant effect of sedation on aerosol production.^1^, ^14^ We also analyze data from aerosol‐producing events using a background subtraction approach described in our previous methodology^1^. Specifically, we consider the following individual aerosol‐generating events: oral extubation, coughing/gagging during the procedure, anesthetic throat spray, and volitional cough.

For the analysis of the inter‐procedure particle counts, there was a mixture of lower‐ and upper‐GI procedures preceding the fallow period (44% lower GI, 56% upper GI). This may affect the concentration of particles at the start of the fallow time: upper GI procedures produce more particles per unit of time, though this may be compensated by the shorter duration (<3x) compared to lower GI procedures.^1^ To avoid this problem, for the inter‐procedure periods we consider the rate of change of particle counts, i.e. the gradients with respect to time. First, we identified all periods when particle count was continuously decreasing (e.g., when no people are present in the room), which we denote as particle ‘sink’ windows, defined when particle concentration decreases for 4 or more consecutive measurements. Across four fallow periods measured in standard endoscopy rooms with portable HEPA filtrations units, we identified 490 such sink windows. As a control, we identified 2305 sink windows across 33 procedures in standard endoscopy rooms without a HEPA filter. Within these sink windows, we compute the rate of clearance of particles and fit an exponential model to determine a dispersal rate constant. We then extrapolate to estimate the time taken to clear 50% of the particles in the room.

### Statistical analysis

All statistical analysis was performed using MATLAB software (The MathWorks, Natick, MA, USA). Building on existing models of respiratory aerosol production we model particle counts using a log‐normal distribution.^15^ For the whole procedure data, a logarithm of the data is first computed (to convert the data from log‐normal to normal) and then a *t‐*test is applied. The resulting means and confidence intervals become ratios when the inverse logarithm (i.e., exponential) is applied to recover raw particle counts. For individual events, the data distribution is modeled as the sum of a log‐normal and normal distribution to account for negative particle counts arising from the subtraction step. A Monte‐Carlo sampling method provides numerical estimates of *p*‐values, mean ratios, and confidence intervals between events.^16^

*A priori* power calculations based on limited previous studies determined that with five replicates per patient, we can detect an effect size (Cohen's d) of 1.98, sufficient to differentiate between a cough and sneeze.^17^, ^18^ We also conducted a retrospective power analysis to indicate what sample sizes may be needed for future studies. For the inter‐procedure analysis, we apply a Mann–Whitney *U* test (due to non‐normality) to establish statistical significance and use bootstrapping (sampling) to obtain confidence intervals.

## RESULTS

The relevant demographics of the patients used in the three different rooms show no statistically significant difference in each characteristic except for body mass index in the Laminar Flow group (Table 1). A comparison of the number of coughs per procedure and coughs per unit of time showed no significant difference. No significant difference in procedure lengths was observed.

**TABLE 1:** Summary table showing demographic data for patients enrolled in this study. *p*‐Values indicate the statistical significance of respective demographic features relative to the conventional ventilation group

**Ventilation** **Scenario variable**	**Conventional ventilation**	**Conventional ventilation + portable HEPA filter**	**Laminar flow theat**
*n*	33	4	4
Age	Range: 24–93	Range: 24–71	Range: 41–74
	Median: 63	Median: 58	Median: 52
		(*p* = 0.359)	(*p* = 0.463)
Sex	Male: 20, Female: 13	Male: 1, Female: 3	Male: 1, Female: 3
		(*p* = 0.542)	(*p* = 0.284)
BMI	Range: 16.3–38.2	Range: 19.8–26.3	Range: 24.9–39.3
	Median: 24.8	Median: 24.8	Median: 32.4
		(*p* = 0.342)	(*p* = 0.014)*
Smoking	Smoker: 8	*Smoker*: 1	*Smoker*: 1
	Non‐smoker: 24	Non‐smoker: 3	*Non‐smoker*: 3
		(*p* = 0.571)	(*p* = 0.571)
Hiatus hernia	Yes: 9, No: 24	Yes: 1, No: 3	Yes: 1, No: 3
		(*p* = 0.585)	(*p* = 0.585)
Sedation	*Midazolam*: 14	Midazolam: 2	Midazolam: 3
	Throat spray only: 19	*Throat spray only*: 2	Throat spray only: 1
		(*p* = 0.560)	(*p =* 0.323)
No. of coughing/gagging events per procedure	Mean: 0.79	Mean: 0.75	Mean: 1.00
		(*p* = 0.722)	(*p* = 0.999)
Procedure duration (minutes)	Mean: 7.4	*Mean: 6.5*	Mean: 5.1
		(*p* = 0.835)	(*p* = 0.280)

**FIGURE 2:**
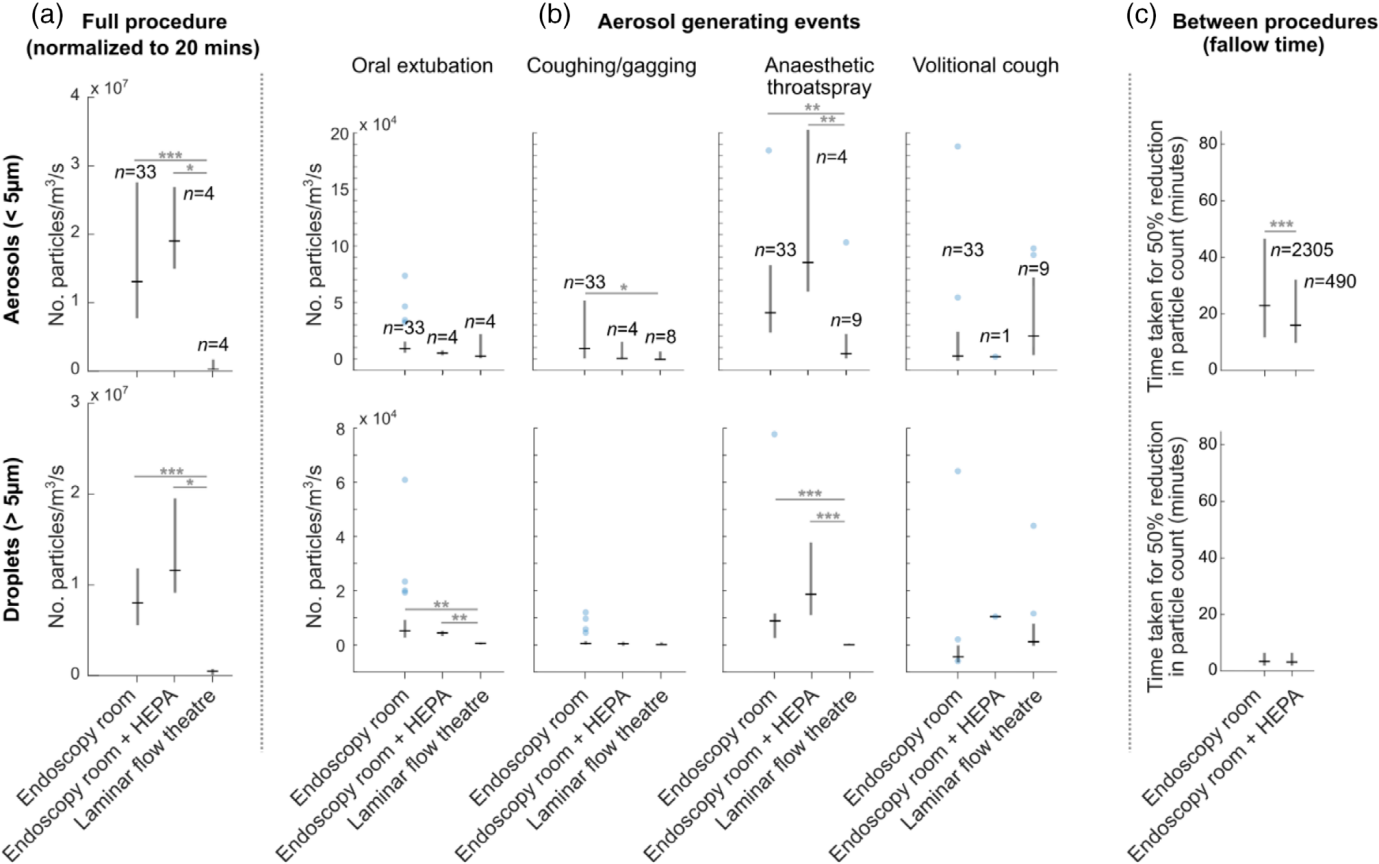
Effect of different room ventilation schemes on aerosol and droplet counts. (a) Total particle counts across whole procedures. (b) Comparison of three aerosol‐generating events. (c) Investigation of particle clearance rate with and without portable high‐efficiency particulate air (HEPA) filter, showing significant speed increase. *p < 0.05, **p < 0.01, and ***p < 0.001.

For the whole procedure analysis (Figure 2a) in the aerosol size range we find no significant reduction in total particle count using the HEPA filtration unit (*p* = 0.50) but a significant reduction when using a laminar flow room compared to standard endoscopy room (28.5x, 95% CI 13.9–58.3, *p* < 0.001) and to standard endoscopy room with portable HEPA filtration unit (37.5x, 95% CI 5.7–245.5, *p* < 0.05). A similar trend is observed for droplets (>5 μm diameter) with a significant reduction in the count for laminar flow theatre compared to the standard endoscopy room (30.7x, 95% CI 16.9–55.9, *p* < 0.001), and standard endoscopy room with HEPA filtration unit (50.0x, 95% CI 10.8–231.4, *p* < 0.001).

We next consider individual aerosols generating events: oral extubation, coughing/gagging anesthetic throat spray, and volitional cough (Figure 2b). For oral extubation, we find that in the aerosol size range particle counts are statistically comparable across the 3 room types, but in the droplet size range particle counts are significantly reduced in the laminar flow room compared to standard endoscopy room (12.2x, 95% CI 5.0–38.3, *p* < 0.01) and to standard endoscopy room with portable HEPA filtration (10.1x, 95% CI 4.0–35.7, *p* < 0.01). The average particle diameter for oral extubation is significantly smaller in laminar flow (0.22 μm) compared to the endoscopy room (2.8μm, p < 0.05) and to the endoscopy room with a portable HEPA filter (4.6 μm, *p* < 0.01). For coughing/gagging in the aerosol size range we measure a significant reduction in laminar flow theatres compared to endoscopy (6.9x, 95% CI 1.22–61.9, *p* < 0.05) but find no significant difference compared to standard endoscopy room with portable HEPA filtration system. In the droplet size range, we find no significant difference between any of the room types, but this may simply reflect the small average particle size of coughing/gagging.^1^ For the application of anesthetic throat spray in the aerosol size range we find a significant reduction in laminar flow theatres compared to standard endoscopy rooms (8.4x, 95% CI 2.03–64.1, *p* < 0.01) and standard endoscopy rooms with portable HEPA filtration (20.7x, 95% CI 2.9–199.3, *p* < 0.01). A similar trend is observed in the droplet size range with laminar flow theatres measuring fewer particles than standard endoscopy rooms (46.0x, 95% CI 7.4–438.6, *p* < 0.01) and standard endoscopy rooms with portable HEPA filtration (169.0x, 95% CI 21.2–1855.3, *p* < 0.001). For volitional coughing, we did not observe a significant reduction (*p* = 0.11) between laminar flow and standard endoscopy rooms (comparison not possible with HEPA filtered room due to only one recorded event).

Finally, for our analysis of particle clearance rates (Figure 2c.) we found that with HEPA filtration the median 50% clearance time was 16.8 min compared to 23.8 min without. Further, we found that the decay rate in the aerosol size range with the HEPA filter is 1.41x faster (*p* < 0.001), implying an effective increase in air change rate from 15–17 to 21–24 ACH. We did not observe any significant reduction for particles in the droplet size range, likely because these particles clear much more quickly due to gravity (median: 3.1 min for 50% clearance). For the laminar flow theatre, the particle counts between procedures are exactly zero due to particle clearance faster than our present experiment can detect.

## DISCUSSION

Overall, we find that the use of laminar flow theatres significantly reduces aerosols and droplets measured near the patient's mouth, typically by a factor of >5x during upper GI endoscopic procedures. This observed reduction also applies to individual aerosol‐generating events (oral extubation, coughing/gagging, application of throat spray), which are significantly reduced in magnitude (>5x). However, respiratory coughing may still pose a risk as this is not significantly reduced. We do not find a significant reduction in particle counts during procedures using portable HEPA filtration units, implying their effect is too small to be measured with our sample size, particularly when used in a room with adequate pre‐existing ventilation. This is expected because these filters are significantly less powerful than the whole‐room ventilation in laminar flow theatres. However, by analyzing fallow periods we find that portable HEPA filtration units can increase aerosol clearance rates by ∼40%, which could reduce the safe fallow time between procedures by 5–7 min.

The demographics of the patient groups for different ventilation conditions are not statistically different except for a small difference in body mass index: however, our previous work found that among these features, only the presence of a hiatus hernia significantly impacts aerosol production so we do not expect this difference in body mass index will have a significant effect, although other studies using different methodologies have found small positive correlations.^1^, ^14^

For oral extubation events, the smaller average particle diameters observed (∼10x smaller) and the lower number of particles in the droplet size range (12.2x less) in the laminar flow ventilation, suggests that this type of ventilation removes larger particles very effectively, either through direct dispersal or evaporation. However, previous aerosolization studies suggest that evaporation would not have a major impact over such short distances of travel (0.1 m) particularly for larger particles.^9^ A similar reduction in particle counts was not, however, observed for volitional coughing events. We hypothesize this may be due to the substantially higher particle velocity for volitional coughing compared to less forceful involuntary gagging, enabling more airborne particles to reach the detector.^9^

There are a few key limitations of this study. The first is a small sample size as there are only four examples each for laminar flow and HEPA‐filtered procedures so only large effects are detectable. Retrospective analysis of study power suggests that, given the measured data, to test the effect of the laminar flow theatre with power 0.9 and *p* = 0.05 would need a sample size of *n* = 3, and a with our actual measured sample size of *n* = 4, study power is >0.999. To test the effect of the portable HEPA air filtration unit with power 0.9 and *p* = 0.05 would require a sample size *n* > 700, which would be challenging to measure given the available numbers of patients. Our conclusion with our smaller sample of *n* = 4 is therefore that the effect size of this HEPA filter is small, significantly less than for the laminar flow theatre. However, for our particle clearance rate analysis, our sample size of *n = 490* and *p = 0.05* gives a study power of 0.997, sufficient for analyzing the effect of the HEPA filter. Larger future studies are needed to quantify the weaker effects of HEPA filtration units on intra‐procedure particle counts. The second limitation is the range of room geometries: we test only a single laminar flow theatre and a single position of the HEPA filtration unit. Because both installations were performed professionally and validated using smoke flow visualization tests and balometers, it is reasonable to assume near‐optimal performance. However, in wider deployment, placement may be more constrained leading to suboptimal performance. Future studies should consider different room geometries, placement of HEPA filtration units, and brands' power ratings. These studies should consider HEPA filtration units in poorly ventilated rooms where they may have a greater impact. Finally, though our study does not directly measure viral content or infectivity so future studies could combine particle counting with air sampling and follow‐up studies of patients to gauge infection levels.

The clinical implications of our findings can be applied both during procedures and to fallow times. Specifically, if a high‐risk or actively infectious patient must undergo an emergency upper GI endoscopy laminar flow theatre should be considered. This is particularly true during disease outbreaks when PPE may be in short supply or testing is not available. For routine patients, rapid testing combined with PPE likely remains the most efficient strategy. In the rooms tested here with relatively good ventilation (15–17 ACH) portable HEPA filtration units seem not to have a large effect on intra‐procedure air quality so may be more useful in rooms with very poor ventilation: further studies are required to confirm this. Therefore, standard precautions with regard to PPE should still be applied.

However, between procedures when the room is empty, we find that particles are cleared up to 40% faster in the presence of a portable HEPA filtration unit. This implies this fallow time could be reduced, for example, from 20 to 13–15 min, while still removing the same quantity of potentially infectious particles. This could increase the throughput of patients back to near‐normal levels and reduce backlogs. We would therefore recommend the routine use of portable HEPA filters to safely reduce the fallow time for rooms with 15–17 ACH or less.

## CONFLICT OF INTEREST STATEMENT

None.

## ETHICS STATEMENT

Wales Research Ethics Committee (IRAS no. 285595).

## PATIENT CONSENT

Obtained

## Data Availability

Data associated with this publication are available at https://doi.org/10.17639/nott.7112. Code used for data analysis in this publication can be found at https://github.com/gsdgordon/aerosols.
